# Important and Notable Gathering

**Published:** 1909-03

**Authors:** 


### EDITORIAL DEPARTMENT.

### IMPORTANT AND NOTABLE GATHERING.

A national conference on criminal law and criminology will be held at the Northwestern University, Chicago, June 7th and 8th, in celebration of the fiftieth anniversary of the founding of the Northwestern University School of Law. A preliminary prospectus has been issued and invitations in the name of President Abram Winegardner Harris, of Northwestern University, jointly with the committee on organization, Roscoe Pound, Professor of Law, chairman, have been sent out to select a list of persons more or less interested in and identified with the subjects named. The list embraces many of the most distinguished names in America, the professions of law, of medicine being mainly represented. It is by no means complete and many new names will be added. It is contemplated to form a permanent national society with branches in each State for the study of criminal law, criminal anthropology, penology, etc. The officers of the Committee on Organization are Roscoe Pound, Chairman; Harvey C. Carbaugh, Vice-Chairman; Nathan W. McChesney and Frederick B. Crossley, Secretaries; Geo. R. Nichols and Harry L. Shaver, Assistant Secretaries. The Executive Sub-Committee consists of Judge Orrin N. Carter, Judge Albert C. Barnes, Dr. Archibald Church, Dr. Henry B. Favill, Prof. Chas. R. Henderson, Robert N. Holt, Frank I. Loesch, John L. Whitman, John H. Wigmore.

The committee, in the preliminary prospectus, announce that "the immediate occasion of the conference is the celebration of the fiftieth anniversary of Northwestern University School of Law. Instead of the conventional exercises upon such occasions, it is desired to signalize the anniversary by a public service—by something of permanent value and general usefulness. Criminal law and preventive, police and penal administration afford a conspicuous opportunity for such a service. Many today are contending that our American criminal law and administration fail adequately to protect society and are ineffective as a corrective system. The importance of investigating these criticisms and the proposed remedies is now generally recognized. Moreover, the sciences of criminal anthropology and sociology are calling for a recasting of ideas the world over, and are making it evident that legislation and legal progress in the future must be founded upon a scientific study and understanding of matters to which the legal profession and the public are only just beginning to give attention. A thorough reconsideration of criminal law and procedure in the light of the several sciences that contribute to criminology must take place in the not-distant future. The best means of furthering this purpose and of insuring that it be undertaken with system and intelligence, would seem to be a National conference, at which ideas may be exchanged, differences among those who approach the subject from divers standpoints may be adjusted, and a propaganda may be organized for the spread of accepted principles and the study of suitable revisions of criminal law and procedure. Such a conference might well end in a National society for these purposes. To these ends it is purposed to bring together in a National conference the leaders among those interested in criminal law, criminal procedure and criminal-law administration, whether as judges, lawyers, prosecutors, prison superintendents, police officers, alienists, criminologists, psychologists or sociologists. It is believed that an immediate effect will be to stimulate public interest in problems of criminology, criminal law and criminal procedure, in itself an important beginning toward a sound basis for legislation and reform."

---

AJAX DEFIES THE LIGHTNING.—Good pluck, perhaps, but bad taste and worse judgment. Our handsome young friend from Pennsylvania, the popular and efficient Secretary of the Texas State Medical Association, who, by virtue of his office graces the tripod of its Journal, hurls a harmless thunderbolt at our devoted head, in a paroxysm of wrath, in defense of his friend, master and prototype, Simmons of the Octopus. He seems to regard Dr. Simmons as synonymous with the American Medical Association, and in the February number of the *State Journal of Medicine* proceeds to defend the American Medical Association against what he calls "Final Attack of the Proprietaries."

No one has attacked the American Medical Association; but the uncontrolled medical press have justly protested against the high-handed and outrageous and extremely offensive course that the man at the helm of the great medico-political machine has pursued with reference to the medical press. Especially when it is remembered that this party is an aline, who a while ago was running a water cure establishment with homoeopathy and liquid oxygen on the side, and advertising the same in the secular press, after the manner of the quack and charlatan, now dictates medical ethics to the regular profession and censors the advertising pages of the journals which rebel against his autocracy, it is especially exasperating. The war is on Simmons, not on the American Medical Association (which in an evil hour fell under the spell of his fanaticism). The mess that he has made of organized medicine would be laughable if it were not so serious. I do not know which sentiment predominates—indignation and contempt—for the manner in which the rank and file, the disfranchised of the profession, were tricked into submitting with a lamb-like docility to being disfranchised and led into a degrading peonage, and the outrageous denunciation of the medical journals as unclean and unworthy of support; or amusement at the farcical yet pitiful attitude the profession has been placed in through the propaganda of unification and mixed boards. I can hardly treat the subject with becoming gravity. I feel disposed to laugh, and every time I think of the *fulmen brutum* hurled at me by my handsome little Yankee friend, Captain of the Hosts, Chief Scribe, Keeper of the Records and Seal and Gatherer of The Tribute, I feel my risibility stirring me to cackle right out.

But, while I do not intend to be put on the defensive by any such clap trap, I would be recreant to my manhood and self-respect, as well as to the medical profession of Texas, who have honored and trusted me, if I did not resent and denounce as false the assertions that "a (sic.) Texas Medical Journal" (meaning THE TEXAS MEDICAL JOURNAL) has been selected by the "association of proprietaries" to open the attack on the American Medical Association, and that "the medical press has been subsidized," etc., and a lot of other stuff as absurd as it is false, as relates to this Journal. I know of no such organization, never had any communication or dealings with any such, or any person claiming to represent such association. The charge to the contrary renders him who makes it eligible to Mr. Roosevelt's famous club. Moreover, the intimation that the "Red Back" can be controlled, dictated to, "selected" or influenced to do aught but defend principle against shenanegan and bossism, would be laughed at by those who know the man behind the pen. No one knows what's coming, and when it comes all run to see where the lightning struck.

What irritated our young friend, gave him vertigo, as it were, and made him so far forget himself as to let his angry passions rise (which he should never do), and what he mistook for men in Lincoln green and men in buckram, was the "Red Back's" burlesque history of the Simmons-McCormick organization, "The Evolution of the Great Medical Trust." It was a veritable shirt of Nessus to his delicate cuticle. He thought it was an attack on the American Medical Association, and, like a faithful partisan, he rushed to the defense of his master. "Who hits Simmons hits Me. Bruhr-r-r!"

Now this may be good pluck, but it is bad taste. It ill becomes a young man, comparatively recently arrived, to thus asperse one who, having assisted in the organization of the first medical college Texas ever had, was teaching medicine when this valiant young Ajax was "puking in his nurse's arms"; a veteran of the medical profession in Texas who has given twenty-five years of his three score and ten to a conscientious and vigorous advocacy and defense of the true interests of legitimate medicine; whose ethics, its honors, its history and traditions he venerates.

Moreover, it is rash, execrable judgment to attack the rock-ribbed, time-tried, fire-tested Old "Red Back." Many have done so before him and got sick.

It reminds me of an anecdote of my dear old friend, the late Governor Hogg. He was making a speech in court and was giving his opponent, a little fellow named Jenkins, particular hell. Jenkins jumped on a chair behind the Governor and began to pound his big fat back with both fists. The big Governor glanced over his shoulder and said: "What you doin', Jenkins?" "I'm a fightin', I 'ganny," said Jenkins. "Sit down, Jenkins, *sit down,*" said the Governor, and he went on with his speech, just as the "Red Back" is going on in its fight for right and principle and the maintenance of the traditions and honor of legitimate medicine.

This assault on the "Red Back" may be illustrated further by another anecdote.

I was going up the Tallahatchie river on a little stern-wheel boat just after the war. The boat stopped at a woodyard to take on some wood. All the passengers went out on deck, and amongst them was a little Yankee just come South. He wore a plaid suit and a checked cap with a button on top, and had an eyeglass stuck in his eye. There stood on the bank near the wood pile a long-legged, knock-kneed, cross-eyed country fellow, a young giant. He was the very picture of rural simplicity as he gazed on in apparent wonder, with his hands stuck into his breeches pocket up to his elbows, coatless and with one gallus. Our little Yankee, with a wink at those around him on the boat, said: "Watch me scare hell out of that fellow." Putting on a fierce look, and with a pistol in one hand and a bowie knife in the other, he rushed up to Simplicus (thinking he would run), exclaiming: "You are the very fellow I've been looking for." Instead of running, Simplicus straightened out his right arm and knocked the dude into the river. Looking up at the assembled passengers on the deck, with a grin that was childlike and bland, he said:

"Anybody else up there lookin' for me?"

---

Dr. Fred J. Mayer, long time Sanitary Inspector of Louisiana and the author of the "Louisiana System of Sanitary Education" so successful in that State, was invited by Mississippi to accept a similar position with the Mississippi Board and to lecture throughout the State on the importance and necessity of sanitary reform. He accepted the position and is doing excellent missionary work, not only in Mississippi but in other States. By invitation of the Texas authorities and the Legislature he delivered an address on the subject before that body February 26 (ult.), and scored. His forceful showing of the needless waste of human life—its economic value, and the saving that would result to the State, treating the subject solely from an economic standpoint—and omitting any considerations of humanity—impressed the large audience of legislators and citizens most profoundly, and it is thought that it will insure the passage of the Board of Health bill now pending. Dr. Mayer is an orator as well as an effective lecturer.

Dr. Mayer was introduced by Dr. Daniel at request of State Health Officer Brumby and the Speaker of the House.

---

The Inalienable Right to "Do So."—The *Lancet Clinic* says, in defense of W. C. L. O. Simmons (Water-Cure-Liquid Oxygen): "If he is a member of a ring seeking to control the Association, the inalienable right of investigation at the next annual meeting is at the disposal of any one wishing to do so (sic.); and the doctor has no such influence as to prevent a combination against him if the facts warranted a searching inquiry."

Hasn't he tho'? At Atlantic City in June, 1908, Dr. H. O. Walker, of Detroit, offered a resolution to appoint a special committee to go over the books of the Secretary-Editor, and it was promptly tabled! "The king can do no wrong." It will thus be seen that no one will be permitted to "do so" without the consent of the ring.

